# Special Issue “SARS-CoV-2 Innate and Adaptive Immune Responses”

**DOI:** 10.3390/v14112363

**Published:** 2022-10-26

**Authors:** Rémi Planes, Elmostafa Bahraoui

**Affiliations:** 1 Institute of Pharmacology and Structural Biology (IPBS), University of Toulouse, CNRS, 31400 Toulouse, France; 2 Institut Toulousain des Maladies Infectieuses et Inflammatoires (INFINITY), INSERM, CNRS, Université Paul Sabatier Toulouse III, 31062 Toulouse, France

Since the end of 2019, humanity has been facing the emergence of a new large positive-sense, single-stranded RNA virus called severe acute respiratory syndrome coronavirus 2 (SARS-CoV-2), which causes a respiratory disease with substantial morbidity and mortality called coronavirus disease 19 (COVID-19). This pandemic has unprecedently mobilized the efforts from worldwide researchers and clinicians in order to better understand the immune mechanisms that control the pathogenicity of SARS-CoV-2 infection. Typically, the infection is associated with two distinct clinical features. Although in most cases (~90%) the infection is asymptomatic or associated with mild symptoms, some patients (~10%) experience more severe disease and develop acute respiratory distress syndrome with systemic inflammation, cytokine storm, tissue damage, thromboembolic complications, and/or cardiac injury, which can be fatal in ~1–2% of cases. While both innate and adaptive arms of the host immune response are crucial in conferring protection or susceptibility to disease, the immunological features of SARS-CoV-2 infections are still poorly understood. In our Special Issue, “SARS-CoV-2 Innate and Adaptive Immune Responses”, we propose a compilation of 13 articles including 4 reviews and 9 original research articles from several disciplines including immunology, virology, biochemistry, and clinical data which address various aspect of anti-SARS-CoV-2 innate and adaptive immune responses. 

The host innate immune response against SARS-CoV-2 infection is initiated by the detection of specific viral features by dedicated sets of innate immune sensors collectively called pattern recognition receptors (PRRs), which trigger the activation of specific genes devoted to orchestrating the eradication of the pathogen; these genes typically encode cytokines, interferons and chemokines. Particularly, the interferon response plays an important role in tackling viral infections. How SARS-CoV-2 and its variants interplay with the interferon response is a central question. The induction of the interferon response and its capacity to control SARS-CoV-2 replication and particularly the Omicron variant has been investigated in [1]. Different aspects of the innate immune sensing mechanism of coronaviruses and viral evasion strategies are reviewed in [2,3]. Crucial immune responses that take place in the upper respiratory tract, the primary entry site of SARS-CoV-2, are reviewed in [4]. Among different PRRs involved in SARS-CoV-2 detection, Planes et al. present a molecular characterization of the interaction between the SARS-CoV-2 Envelope (E) protein and TLR2 [5]. In addition to the TLR2 pathway, SARS-CoV-2 infection modulates the expression of various cellular genes with important implications in inflammation and tissue/organ dysfunctions, which were explored in [6]. With the aim of characterizing new genetic biomarkers of COVID-19 severity, Zanchettin et al. examined the presence of gene polymorphism in COVID-19 patients who succumbed to severe COVID-19. The authors found a potential association with an allelic variant already described in other inflammatory diseases of macrophage activation syndrome (MAS) pathway [7]. Other important actors of anti-SARS-CoV-2 innate immune response are the cytotoxic cells including natural killer (NK) cells, which eliminate infected cells and interact with various other important immune cells. Moraga et al. proposes an important role of the alteration of NK-cell-mediated immunity in COVID-19 disease [8].

In addition to innate immunity, the adaptive immunity executed by cytotoxic CD8+ T cells and antibody-producing B cells and orchestrated by CD4+ helper T cells plays a pivotal role in the establishment of long-term acquired immunity. One response of particular importance is the production of neutralizing antibodies targeting spike protein from SARS-CoV-2. While serological assays are broadly used to track seroconversion in response to vaccination or natural infection, such assays are not informative about the presence of antibodies with neutralizing capacity. To circumvent this limitation, Phelan et al. describe an innovative method to evaluate the presence of anti-SARS-CoV-2 antibodies with neutralization capacity in serum samples of COVID-19 patients using an adapted ELISA assay [9]. Although adaptive immunity is an essential weapon against SARS-CoV-2 infection, understanding the impact of pre-established diseases and/or the use of certain specific treatments on the development of adaptive immunity in response to vaccination or natural infection is a central question that was explored in two research articles of this issue [10,11]. Drug repurposing is an artful strategy to screen available and efficient treatment against emerging diseases. However, how such treatment affects the development of adaptive immunity and protection from reinfection warrant extensive studies. In this context, Shinada et al. explored the effect of favipiravir treatment of COVID-19 patients with promising results [12]. One important concern is the emergence of viral variants with enhanced pathogenicity. Different aspects of the target cell virus interaction that guide the evolution of new variants are discussed [13].

The editors would like to thank all the authors for their contribution to this Special Issue. These works may help to provide a better understanding of the genetic, cellular, and viral determinants of the anti-SARS-CoV-2 immune response and could contribute to the development of new therapeutic approaches.
